# Continuous association of total bile acid levels with the risk of small for gestational age infants

**DOI:** 10.1038/s41598-020-66138-y

**Published:** 2020-06-09

**Authors:** Li Li, Wei Chen, Li Ma, Zhi Bing Liu, Xue Lu, Xing Xing Gao, Yan Liu, Hua Wang, Mei Zhao, Xiao Lan Li, Lin Cong, De Xiang Xu, Yuan Hua Chen

**Affiliations:** 1 School of Basic Medical Sciences, Anhui Medical University, Hefei, 230032 China; 2 Department of Obstetrics and Gynecology, First Affiliated Hospital of Anhui Medical University, HeFei, 230022 China; 3 School of Public Health, Anhui Medical University, Hefei, 230032 China; 4 School of Nursing, Anhui Medical University, Hefei, 230032 China; 5 Implantation and Placental Development Laboratory, Centre for Reproductive Health, Hudson Institute of Medical Research, Clayton, VIC Australia

**Keywords:** Signs and symptoms, Risk factors

## Abstract

The association between maternal serum total bile acid (TBA) levels and small-for-gestational-age (SGA) infants is unclear. We investigated the association between various degrees of serum TBA levels and the risk of SGA infants in a Chinese population. The current study performed a cohort study among 11811 mothers with singleton pregnancy. Subjects were divided into seven categories according to maternal serum TBA levels. Interestingly, birth sizes were reduced, whereas the rate of SGA infants was increased across increasing categories of serum TBA. Compared to category 1, adjusted ORs (95%CI) for SGA infants were 0.99 (0.82–1.21) in category 2, 1.22 (0.97–1.53) in category 3, 1.99 (1.53–2.58) in category 4, 2.91 (2.16–3.93) in category 5, 4.29 (3.33–5.54) in category 6, and 9.01 (5.99–13.53) in category 7, respectively. Furthermore, adjusted ORs (95%CI) for SGA infants for each 1-SD increase in serum TBA levels were 1.36 (1.29–1.43) among all subjects, 2.40 (1.82–3.45) among subjects without cholestasis, and 1.13 (1.06–1.22) among subjects with cholestasis, respectively. These results suggest that gestational cholestasis increases the risk of SGA infants. Additionally, our results indicate strong, continuous associations of serum TBA levels below those diagnostic of cholestasis with a decreased birth sizes and an increased risk of SGA infants.

## Introduction

Intrahepatic cholestasis of pregnancy (ICP), also named gestational cholestasis, is defined as the presence of pruritus in combination with elevated serum total bile acid (TBA) levels (≥10 μmol/L). ICP is one of the most prevalent obstetric disease^1,2^. ICP occurs usually in the second half of pregnancy until delivery. The incidence of ICP ranges from 0.4% to 15% in different countries, ethnic populations and climatic conditions^3,4^. The majority of studies had demonstrated that ICP was associated with adverse maternal outcomes, including 3-fold increased risks of gestational diabetes mellitus and pre-eclampsia^5–7^. A large cohort study from Sweden showed that women with ICP had increased risks of later liver and biliary tree cancer, later specifically diabetes mellitus, later autoimmune-mediated and cardiovascular diseases after childbirth^8^. On the other hand, several epidemiological studies reported the association between ICP and the increased risks of adverse fetal outcomes, including spontaneous and iatrogenic preterm delivery, a low (<7) 5-minute Apgar score, respiratory distress syndrome, meconium-stained fluid, stillbirth and intrauterine fetal death^3,9–11^. In addition, a report on human and rodent animal demonstrated that ICP was also associated with sex-specific increased susceptibility to severe obese, diabetic phenotype with hepatosteatosis in adult offspring, indicating a programming effect of the high bile acid exposure in utero^12,13^^.^

Small for gestational age (SGA), defined as fetal weight less than the 10th percentile based on gender and gestational age, is one of the leading causes for stillbirth, neonatal death and perinatal morbidity^14–16^. Several epidemiological reports showed that the risks of autism in childhood and cardiovascular and metabolic diseases in adulthood were increased in people born with SGA^17–20^. Nevertheless, no report analyzed the association between ICP and an increased risk of SGA infants in a cohort study. It is more obscure whether maternal serum TBA levels less severe than that in cholestasis are associated with an increased risk of SGA infants.

The present study conducted a birth cohort study to investigate the risk of SGA infants associated with various degrees of serum TBA levels. The present study found that ICP elevated the risk of SGA infants. Additionally, our results indicate strong, continuous associations of serum TBA levels below those diagnostic of cholestasis with a decreased birth sizes and an increased risk of SGA infants.

## Results

### The demographic characteristics and laboratory measurements of study participants

The demographic characteristics of study participants were presented in Table 1. There were significant differences on maternal age, education, and mode of delivery among different groups (Table 1). No significant differences were observed on maternal pre-pregnancy BMI, parity, and gravidity among different groups (Table 1). The incidence of preeclampsia was significantly lower in the TBA <10 μmol/L group than those in the other two groups (Table 1). No significant differences were observed on the incidence of pregnancy-induced hypertension and gestational diabetes mellitus among different groups (Table 1). Maternal serum alanine transaminase concentrations, aspartate transaminase concentrations, serum total bilirubin concentrations, direct bilirubin concentrations, and indirect bilirubin concentrations were measured. Results showed that those were increased across the increasing serum TBA levels categories (Table 2).

**Table 1:** Characteristics of the study participants.

Demographic variables	Serum TBA levels (μmol/L)			P-value
	<10.0 (n = 11120)	10.0–39.9 (n = 563)	≥40.0 (n = 128)	
Maternal age (years)				
<25 [n (%)]	1636 (14.71)	121 (21.49)	37 (28.91)	<0.001
25–34 [n (%)]	8227 (73.98)	371 (65.90)	71 (55.47)	
≥35 [n (%)]	1257 (11.30)	71 (12.61)	20 (15.63)	
Pre-pregnancy BMI (kg/m^2^)				
<18.5 [n (%)]	1916 (17.23)	113 (20.07)	29 (22.66)	0.159
18.5–22.9 [n (%)]	6875 (61.83)	328 (58.26)	79 (61.72)	
23.0–27.4 [n (%)]	2003 (18.01)	102 (18.12)	15 (11.72)	
≥27.5 [n (%)]	326 (2.93)	20 (3.55)	5 (3.90)	
Maternal education (years)				
≤9 (Junior school)	3547 (31.72)	288 (52.93)	74 (57.81)	<0.001
10–15 (High school)	3477 (31.09)	155 (26.47)	31 (24.22)	
≥16 (University)	3689 (32.99)	111 (19.00)	15 (11.72)	
Data missing	407 (4.20)	9 (1.60)	8 (6.25)	
Mode of delivery [n (%)]				
Vaginal delivery	6276 (56.44)	342 (60.75)	81 (63.28)	0.042
Cesarean delivery	4844 (43.56)	221 (39.25)	47 (36.72)	
Parity [n(%)]				
1	8288 (74.53)	418 (74.25)	89 (69.53)	0.432
≥2	2832 (25.47)	145 (25.75)	39 (30.47)	
Gravidity				
1	5931 (53.34)	297 (52.75)	61 (47.66)	0.428
≥2	5189 (46.66)	266 (47.25)	67 (52.34)	
Gestational diabetes mellitus [n(%)]				
Yes	917 (8.25)	60 (10.66)	12 (9.38)	0.121
No	10203 (91.75)	503 (89.34)	116 (90.62)	
Gestational hypertension [n(%)]				
Yes	359 (3.23)	21 (3.73)	8 (6.25)	0.135
No	10761 (96.77)	542 (96.27)	120 (93.75)	
Preeclampsia [n(%)]				
Yes	611 (5.49)	77 (13.68)	18 (14.06)	<0.001
No	10506 (94.51)	489 (86.32)	110 (85.94)	

Abbreviation: TBA, total bile acid.

**Table 2:** Laboratory measurements within the study participants.

Parameter	Serum TBA levels (μmol/L)						
	<2.0 (n = 3196)	2.0–3.9 (n = 4580)	4.0–5.9 (n = 2075)	6.0–7.9 (n = 835)	8.0–9.9 (n = 434)	10.0–39.9 (n = 563)	≥40.0 (n = 128)
Alanine transaminase (IU/L)	13.7 ± 14.7	17.0 ± 27.5**	21.1 ± 34.1**	29.4 ± 49.9**	34.4 ± 64.1**	110.2 ± 152.6**	189.3 ± 171.4**
Aspartate transaminase (IU/L)	20.3 ± 13.2	21.8 ± 18.4	25.0 ± 31.2**	30.1 ± 40.2**	36.2 ± 65.1**	93.2 ± 128.8**	168.7 ± 167.0**
Total bilirubin (μmol/L)	8.1 ± 3.4	8.1 ± 11.1	7.7 ± 3.8	8.0 ± 4.1	8.7 ± 4.7	12.6 ± 11.4**	33.9 ± 68.7**
Direct bilirubin (μmol/L)	1.7 ± 0.9	1.8 ± 1.9	2.0 ± 4.2	2.1 ± 1.9	2.5 ± 4.3*	7.5 ± 5.2**	24.4 ± 33.8**
Indirect bilirubin (μmol/L)	6.7 ± 2.8	6.4 ± 2.9*	6.3 ± 2.9*	6.6 ± 3.3	6.5 ± 3.6	4.1 ± 5.8**	9.5 ± 10.3**

Abbreviation: TBA, total bile acid.

The mean differences between two groups were analyzed using least significant difference (LSD) post hoc test.

*P < 0.05, **P < 0.01 as compared with < 2.0μmol/L group.

### Birth sizes among different groups

Subjects were divided into seven categories according to maternal serum TBA levels. Birth weight was compared among seven categories. As shown in Table 3, birth sizes, including birth weight, birth length, head circumference and chest circumference, were decreased across increasing categories of serum TBA levels. Gestational age was also compared among seven categories. Gestational age was reduced across increasing categories of serum TBA levels (Table 3).

**Table 3:** Birth sizes and gestational age in different categories.

Parameter	Maternal serum TBA levels (μmol/L)						
	<2.0 (n = 3196)	2.0–3.9 (n = 4580)	4.0–5.9 (n = 2075)	6.0–7.9 (n = 834)	8.0–9.9 (n = 434)	10.0–39.9 (n = 563)	≥40.0 (n = 128)
Birth weight (g)							
Mean ± SD^a^	3288.5 ± 652.5	3213.3 ± 563.7**	3186.7 ± 602.0**	2966.2 ± 650.7**	2776.6 ± 755.0**	2847.6 ± 704.4**	2575.1 ± 685.8**
Median (25^th^, 75^th^)^b^	3310 (3000, 3700)	3300 (3000, 3550)**	3250 (2950, 3550)**	3000 (2650, 3400)**	2900 (2280, 3350)	2900 (2450, 3350)**	2550 (2100, 3000)**
Birth length (cm)							
Mean ± SD^a^	50.1 ± 3.3	49.9 ± 3.1**	49.8 ± 3.1**	48.9 ± 3.5**	47.9 ± 4.5**	48.3 ± 4.0**	47.1 ± 4.0**
Median (25^th^, 75^th^)^b^	50.0 (49.0, 52.0)	50.0 (49.0, 52.0)**	50.0 (49.0, 51.0)**	50.0 (48.0, 51.0)**	49.0 (46.0, 51.0)	49.0 (47.0, 51.0)**	48.0 (45.3, 50.0)**
Head circumference (cm)							
Mean ± SD^a^	33.7 ± 2.3	33.4 ± 2.0**	33.5 ± 2.2**	32.9 ± 2.4**	32.5 ± 2.7**	32.3 ± 2.5**	31.5 ± 2.8**
Median (25^th^, 75^th^)^b^	34.0 (32.0, 35.0)	34.0 (32.0, 35.0)**	34.0 (32.0, 35.0)**	33.0 (32.0, 34.0)**	33.0 (31.0, 34.0)	33.0 (31.0, 34.0)**	32.0 (30.0, 33.0)**
Chest circumference (cm)							
Mean ± SD^a^	33.5 ± 2.6	33.1 ± 2.3**	33.1 ± 2.4**	32.3 ± 2.7**	31.8 ± 3.3**	31.8 ± 2.9**	31.0 ± 2.9**
Median (25^th^, 75^th^)^b^	34.0 (32.0, 35.0)	33.0 (32.0, 34.0)**	33.0 (32.0, 34.0)**	33.0 (31.0, 34.0)**	32.0 (30.0, 34.0)	32.0 (30.0, 34.0)**	31.0 (29.0, 33.0)**
Gestational age (wks)							
Mean ± SD^a^	38.9 + 2.3	39.0 + 2.4	38.8 + 2.5	38.2 + 2.6**	37.6 + 3.1**	37.8 + 2.7**	36.6 + 2.7**
Median (25^th^, 75^th^)^b^	39.4 (38.4, 40.3)	39.4 (38.4, 40.3)	39.3 (38.3, 40.3)	38.9 (37.1, 40.0)**	38.4 (35.8, 39.9)**	38.3 (36.7, 39.6)**	37.0 (35.1, 38.3)**

Abbreviation: TBA, total bile acid; SD, standard deviation.

^a^The mean differences between two groups were analyzed using least significant difference (LSD) post hoc test.

^b^The median differences were analyzed using non-parametric statistics.

**P < 0.01 as compared with < 2.0μmol/L group.

### Association between serum TBA as a categorical variable and the risk of SGA infants

**Figure 1:**
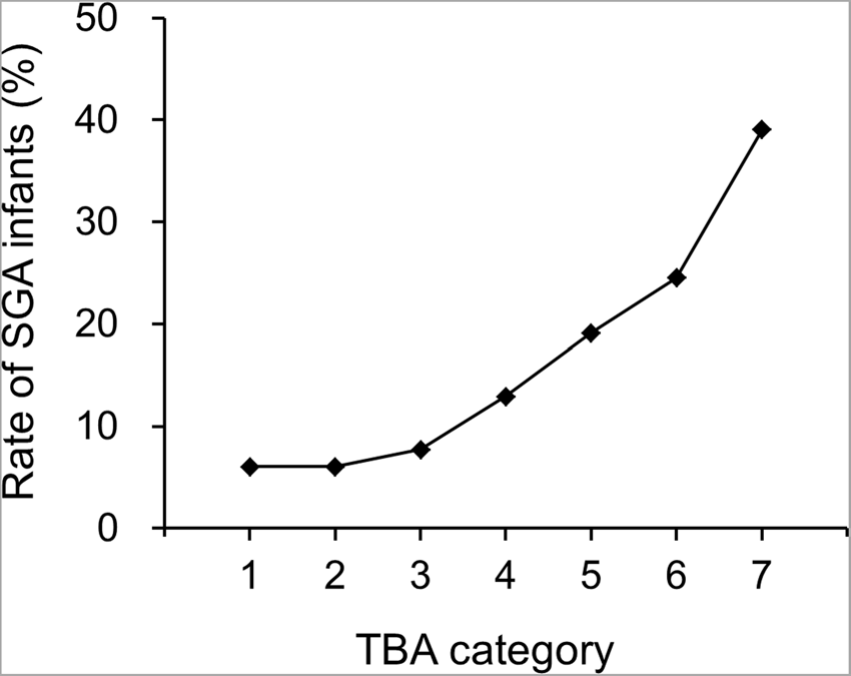
Rate of SGA infants. Serum TBA categories are as follows: category 1, less than 2.0 μmol/L; category 2, 2.0 to 3.9 μmol/L; category 3, 4.0 to 5.9 μmol/L; category 4, 6.0 to 7.9 μmol/L; category 5, 8.0 to 9.9 μmol/L; category 6, 10.0 to 39.9 μmol/L; category 7, 40 μmol/L or more. SGA, small for gestational age; TBA, total bile acid.

Participants were divided into seven categories according to maternal serum TBA levels. The rate of SGA infants across serum TBA levels categories is shown in Fig. 1. With increasing categories of maternal serum TBA levels, the rate of SGA infants was increased (Fig. 1). Table 4 shows the associations of maternal serum TBA levels as a categorical variable with each primary outcome, including odds ratios (ORs) and 95% confidence intervals (95%CIs) for each category, as compared with the lowest category. After adjustment for confounders, there were strong associations with SGA infants that increased across increasing categories of serum TBA levels. Additionally, there were no obvious thresholds at which risk increased (Table 4).

**Table 4:** Crude and adjusted ORs for the associations between serum TBA as a categorical variable and SGA infants.

Parameter	Pregnant women (n)	SGA (n)	Crude OR (95% CI)	Adjusted OR (95% CI) ^a^	Adjusted OR (95% CI) ^b^
Serum TBA levels (μmol/L)					
<2.0	3196	193	1.00	1.00	1.00
2.0–3.9	4580	278	1.01 (0.83, 1.22)	1.02 (0.85, 1.24)	0.99 (0.82, 1.21)
4.0–5.9	2075	160	1.30 (1.05, 1.62)*	1.31 (1.06, 1.63)*	1.22 (0.97, 1.53)
6.0–7.9	835	108	2.31 (1.80, 2.97)**	2.32 (1.81, 2.97)**	1.99 (1.53, 2.58)**
8.0–9.9	434	83	3.68 (2.78, 4.87)**	3.64 (2.75, 4.82)**	2.91 (2.16, 3.93)**
10.0–39.9	563	138	5.05 (3.97, 6.43)**	5.10 (4.00, 6.49)**	4.29 (3.33, 5.54)**
≥40.0	128	50	9.98 (6.80, 14.64)**	9.90 (6.74, 14.55)**	9.01 (5.99, 13.53)**

Abbreviation: TBA, total bile acid; SGA, small for gestational age; OR, odds ratio.

^a^Adjustment for gestational hypertension.

^b^Adjustment for maternal age, pre-pregnancy BMI, maternal education, parity, gestational diabetes mellitus, gestational hypertension and preeclampsia.

**P < 0.01 as compared with < 2.0 μmol/L group.

### Association between serum TBA as a continuous variable and the risk of SGA infants and birth sizes

Table 5 shows the association between serum TBA as a continuous variable and the risk of SGA infants. Adjusted ORs for SAG infants for each 1-SD increase in serum TBA level were 1.36 (95%CI: 1.29, 1.43) among all subjects, 2.40 (95%CI: 1.82, 3.45) among subjects without cholestasis (TBA <10.0 μmol/L), and 1.13 (95%CI: 1.06, 1.22) among subjects with cholestasis (TBA ≥10.0 μmol/L), respectively (Table 5).

**Table 5:** Association between serum TBA as a continuous variable and the risk of SGA infants.

Parameter	Crude models		Adjusted models^b^		Adjusted models^c^	
	OR (95%CI)^a^	*p*	OR (95%CI)	*p*	OR (95%CI)	*p*
TBA category^d^						
All	1.37 (1.31, 1.43)	<0.001	1.37 (1.31, 1.43)	<0.001	1.36 (1.29, 1.43)	<0.001
<10.0 μmol/L	3.41 (2.68, 4.34)	<0.001	3.36 (2.64, 4.28)	<0.001	2.40 (1.82, 3.45)	<0.001
≥10.0 μmol/L	1.10 (1.04, 1.16)	0.001	1.10 (1.04, 1.16)	0.001	1.13 (1.06, 1.22)	0.001

Abbreviation: TBA, total bile acid; SGA, small for gestational age; OR, odds ratio.

^a^ORs were for an increase in serum TBA level of 1 SD

^b^Adjustment for gestational hypertension.

^c^Adjustment for maternal age, BMI, parity, maternal education, gestational diabetes mellitus, gestational hypertension and preeclampsia.

## Discussion

The aim of the present study was to clarify the risk of SGA infants associated with various degrees of serum TBA levels, especially less severe than that in overt cholestasis in a birth cohort study. The present study found that birth sizes, including birth weight, birth length, head circumference and chest circumference, were decreased across increasing categories of serum TBA levels. The association between serum TBA and the risk of SGA infants was analyzed. After adjustment for confounders, there were strongly associations with SGA infants that increased across the increasing serum TBA levels categories.

Maternal demographic characteristics, such as maternal age, pre-pregnancy BMI, parity and maternal education, were associated with birth weight and the risk of SGA infants. A number of epidemiological studies demonstrated that advanced maternal age, primiparity and low BMI before pregnancy elevated the risks of SGA and low birth weight infants^21–23^. Several reports indicated that the risk of SGA was higher in low educational subjects compared with high educational subjects^24,25^. On the other hand, pregnancy complications, such as gestational diabetes mellitus, gestational hypertension and pre-eclampsia, were also associated with birth weight and the risk of SGA. Several reports showed that gestational hypertension and pre-eclampsia elevated the risk of SGA infants^26,27^. In contrast, gestational diabetes mellitus was significantly associated with higher birth weight and 2-fold increased risk of large for gestational age (LGA) infants and macrosomia^28,29^. The present study further estimated the adjusted ORs with 95%CI with respect to the incidence of SGA infants using multiple logistic regression models. After adjustment for these confounders, our results still found that the risk of SGA infants was increased across the increasing serum TBA levels categories.

The mechanism by which elevated serum TBA increases the risk of SGA remains obscure. Several case-control studies showed that the levels of proinflammatory cytokines and chemokines in placenta and maternal serum were significantly higher in the cholestasis group as compared to the control group^30,31^. Reports *in vivo* and *in vitro* found that bile acids stimulated the expression of a series of inflammatory cytokines and reactive oxygen species via activating both signal 1 and 2 of the NLRP3 inflammasome and NF-κB pathway^32–34^. These studies indicated that cholestasis was associated with inflammation and oxidative stress. Indeed, many epidemiological studies showed that maternal serum and umbilical cord serum TNF-α, C-reactive protein and IL-8 levels were significantly higher in the SGA group than in the control group^35,36^. According to a recent nest case-control study, strongly nuclear NF-κB p65 immunoreactivity was observed in placentas from pregnant women with SGA infants^37^. Animal experiments also found that maternal inflammation and oxidative stress resulted in FGR in rodents^38,39^. Therefore, we guess that inflammation and oxidative stress may play a vital role in TBA-mediated SGA. On the other hand, recent evidence suggested that the deficiency or downregulation of selective miRNA may be involved in placental-induced diseases, such as pre-eclampsia and fetal growth restriction, through the epigenetic mechanism^40–42^. Indeed, several studies found that bile acid, such as deoxycholic acid, inhibited miRNA expression in cell lines^43,44^. Consequently, we speculate downregulation of miRNA in placentas may be play a key role in TBA-mediated SGA. Moreover, a recent study reported that maternal serum TBA levels at diagnosis and at delivery were correlated positively with umbilical cord blood TBA levels, which provides evidence that bile acids could transport across the placenta^45^. Recently, numerous reports found that bile acids induced oncosis, necrotic cell death and apoptosis^46,47^. Thus, the present study does not exclude that elevated TBA-associated SGA is due to the direct toxic effect of bile acids.

The present study laid emphasis on whether serum TBA levels less severe than that in cholestasis was associated with an increased risk of SGA infants. However, the present study has three faults. Firstly, the nutritional status, drinking and smoking during pregnancy could affect fetal growth, but we did not have data on the variable. Secondly, the present cohort included only Chinese population, so our results should be treated cautiously when branched out to other ethnic populations. Another potential fault is the lack of information on treatment to pregnant women with cholestasis. Although previous reports demonstrated that treatment with ursodeoxycholic acid, a common drug for treating cholestasis during pregnancy, did not reduce adverse perinatal outcomes in pregnant women with ICP, it was associated with the reduction of serum TBA levels in ICP patients^48,49^.

In summary, the present study investigated the risk of SGA infants associated with various degrees of serum TBA levels in a large birth cohort study. The present study demonstrated that birth sizes were decreased across increasing categories of serum TBA levels. Further analysis found that ICP elevated the risk of SGA infants. Additionally, our results indicate strong, continuous associations of serum TBA levels below those diagnostic of cholestasis with a decreased birth sizes and an increased risk of SGA infants. There were no obvious thresholds at which risk increased. Thus, our study suggests the need to reconsider current criteria for diagnosing and treating ICP.

## Subjects and Methods

### Cohort study

We conducted a retrospective birth cohort in Hefei, a city of central China^21^. Total 13801 pregnant women who delivered at First Affiliated Hospital of Anhui Medical University between January 2011 and December 2014 were recruited. Maternal demographic characteristics and obstetric records were recorded by midwives on the Birthing Outcomes System and all data included in the study was extracted from this database. Maternal nonfasting blood samples were obtained before labor. The exclusion criteria of the current study included the following: unavailable data of detailed delivery records (n = 897), fetal deaths or stillbirths (n = 270), pregnant women giving birth to multiple births (n = 294), induced-abortions (n = 147) and unavailable serum TBA data (n = 382). Finally, 11811 (85.6%) mothers with singleton pregnancy were eligible for this study. The present study obtained ethics approval from the ethics committee of Anhui Medical University (No. 20160010). All participants signed a written informed consent for this study. All methods were carried out in accordance with the approved guidelines.

### Measurement of serum TBA

Serum TBA levels were measured using enzymatic cycling method by an automatic biochemical analyzer (Dirui CS-T300, Ltd, Changchun, China) according to a previous protocol^50^.

### Definition of small-for-gestational age

The cutoff value used for defining the small-for-gestational age (SGA) is birth weight of live-born infants below the 10^th^ percentile for gender and gestational age from a reference population for Chinese^51^.

### Statistical analysis

SPSS 17.0 was used to analysis the data. The mean differences were analyzed using one-way ANOVA and least significant difference (LSD) post hoc test. Categorical variables were analyzed using χ2 tests. The median differences were analyzed using non-parametric statistics (Mann-Whitney U test). The incidence and odds ratio (OR) of SGA infants were calculated in different groups. Multiple logistic regression models were used to estimate the risks of SGA infants in relation to lowest TBA category by crude and adjusted ORs with 95% confidence intervals (95% CI). Linear regression was used to explore the association between serum TBA levels and birth sizes. A *p*-value of <0.05 (two-tailed) or a 95%CI not including 1 and 0 (for relative risk) was considered statistically significant.
